# Human Chorionic Gonadotrophin as a Possible Mediator of Leiomyoma Growth during Pregnancy: Molecular Mechanisms

**DOI:** 10.3390/ijms18092014

**Published:** 2017-09-20

**Authors:** Veronica Sarais, Greta Chiara Cermisoni, Matteo Schimberni, Alessandra Alteri, Enrico Papaleo, Edgardo Somigliana, Paola Vigano’

**Affiliations:** 1 Centro Scienze Natalità, IRCCS San Raffaele Scientific Institute, 20132 Milan, Italy; sarais.veronica@hsr.it (V.S.); matteo.schimberni@gmail.com (M.S.); papaleo.enrico@hsr.it (E.P.); 2 Division of Genetics and Cell Biology, IRCCS San Raffaele Scientific Institute, 20132 Milan, Italy; cermisoni.gretachiara@hsr.it (G.C.C.); alteri.alessandra@hsr.it (A.A.); 3 Fondazione IRCCS Ca’ Granda, Ospedale Maggiore Policlinico AND Università degli Studi di Milano, 20122 Milan, Italy; dadosomigliana@yahoo.it

**Keywords:** human chorionic gonadotropin, fibroid, leiomyoma, estrogen, progesterone

## Abstract

Uterine fibroids are the most common gynecologic benign tumors. Studies supporting a strong pregnancy-related growth of leiomyomas generally claimed a crucial role of sex steroid hormones. However, sex steroids are unlikely the unique actors involved as estrogen and progesterone achieve a pick serum concentration in the last trimester while leiomyomas show a typical increase during the first trimester. Given the rapid exponential raise in serum human Chorionic Gonadotrophin (hCG) at the beginning of gestation, we conducted a review to assess the potential role of hCG in the striking growth of leiomyomas during initial pregnancy. Fibroid growth during initial pregnancy seems to correlate to the similar increase of serum hCG levels until 12 weeks of gestation. The presence of functional Luteinizing Hormone/human Chorionic Gonadotropin (LH/hCG) receptors was demonstrated on leiomyomas. In vitro treatment of leiomyoma cells with hCG determines an up to 500% increase in cell number after three days. Expression of cyclin E and cyclin-dependent kinase 1 was significantly increased in leiomyoma cells by hCG treatment. Moreover, upon binding to the receptor, hCG stimulates prolactin secretion in leiomyoma cells, promoting cell proliferation via the mitogen-activated protein kinase cascade. Fibroid enlargement during initial pregnancy may be regulated by serum hCG.

## 1. Introduction to Leiomyoma Pathophysiology

Leiomyomas or fibroids represents the most common benign tumors of the genital tract in reproductive age women and are associated with significant morbidity and a decline of the quality of life [1]. They mainly consist in collagen-rich masses of proliferating smooth muscle cells with a pseudocapsule but their histological appearance differs from the adjacent normal myometrium since, in most of cases, they demonstrate much more extracellular matrix (ECM) deposition (50% more collagen than normal myometrium with an increased proportion of type I–III). Based on their location and size, leiomyomas can be ulcerated, hemorrhagic, with necrotic and cystic degeneration and, sometimes, becomes calcified [2].

Depending on the study population considered or the diagnostic techniques applied, between 5% and 77% of women were shown to have leiomyomas [3,4]. Leiomyoma prevalence is lower in Europe than in the United States probably because of racial differences. Data on epidemiologic factors associated with leiomyoma risk are only partly elucidated. They include age, race, body mass index (BMI), parity, environmental factors, lifestyles including diet, caffeine and alcohol consumption, smoking, physical activity and stress and other pathologies such as hypertension and infections. The reported impacts of these factors in literature are conflicting [5,6]. Both hormonal and non-hormonal mechanisms may explain the association between these factors and leiomyoma development [5,6,7,8].

Importantly, at least some leiomyomas have a genetic etiology as about 40% of them have been shown to bear genetic alterations [9] and high-throughput sequencing studies have identified recurrent mutations in a limited number of key genes [10]. The most common driver genes involved in the alterations include high mobility group AT-hook 2 (*HMGA2*), mediator complex subunit 12 (*MED12*), fumarate hydratase (*FH*) and collagen type IV (*COL4A5-COL4A6*).

As shown by both clinical and experimental studies, leiomyoma development and growth depend upon ovarian steroids but how they exactly influence these phenomena is not yet fully understood. In line, leiomyomas affect women only during the reproductive period [11,12]. On the other hand, other mediators are thought to affect the growth of uterine fibroids, and among other hormones, human Chorionic Gonadotrophin (hCG) may play a contributing role [13,14]. Human Chorionic Gonadotropin is initially produced by the blastocyst, with RNA already expressed from the eight-cell stage embryo and levels detected in the maternal plasma around the day of implantation. Blastocyst, and later placental hCG is well recognized as a luteo-trophic factor able to maintain progesterone production by the corpus luteum via activation of the Luteinizing Hormone/human Chorionic Gonadotropin (LH/hCG) receptor for the first 3–4 weeks of pregnancy [15].

**Figure 1:**
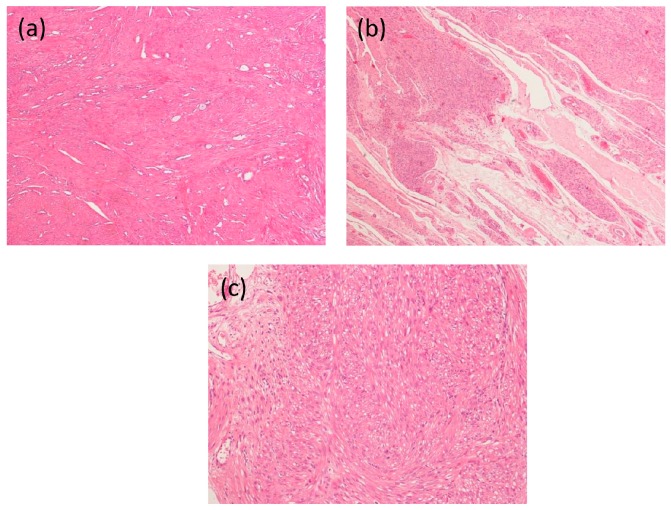
Histological patterns of leiomyoma in the pregnant uterus: (**a**) photomicrograph of non-pregnant normal myometrium; (**b**) photomicrograph of a leiomyoma with extensive mixo-edematous changes in a pregnant woman; and (**c**) photomicrograph of a leiomyoma in a pregnant woman with a cellular area constituted by smooth muscle cells with cytoplasmic vacuoles. Stained with hematoxylin and eosin. Magnification: (**a**,**b**) 125×; and (**c**) 250×.

The prevalence of uterine leiomyomas in pregnant women has been reported to range from 0.1% to 11% [16,17]. Fibroids in pregnancy may undergo significant changes. In particular, hemorrhage, necrosis and degeneration are frequent, occurring in about half of the cases (Figure 1) [18]. Most importantly, pregnancy may boost their growth. Even if available evidence on this issue is not fully consistent [13,16,17,19,20,21,22,23], it is noteworthy that more recent and better-designed studies generally report a nonlinear increase in dimensions, with more growth in the first half of pregnancy, particularly during the first trimester [14]. Considering the increasing incidence of uterine fibroids during pregnancy and the possible correlation between fibroid size and adverse obstetric events [19], it might be important to obtain further information about the regulation of growth of these tumors during pregnancy. Of potential high relevance here is the role of hCG, as this hormone has an exponential raise at the beginning of pregnancy, i.e., during the period of potential maximal fibroid growth. On these bases, we deem of interest performing a comprehensive review aimed at elucidating the potential role of hCG on leiomyoma growth during early pregnancy. Noteworthy, a more in-depth understanding of the hCG-mediated effects might also open a new avenue of pharmacological research, potentially leading to the development of new therapeutic agents.

## 2. Pattern of Growth of Uterine Leiomyomas during Pregnancy

As alluded above, several reports have been published with the aim to clarify the influence of pregnancy on uterine leiomyomas but conclusions are not fully consistent [13,16,17,19,20,21,22,23]. The controversies among studies investigating the effects of pregnancy on fibroid growth can be explained by differences in the study design, the population studied and timing of ultrasound evaluations [13,16,17,19,20,21,22,23]. Noteworthy, some contributions have assessed leiomyoma increase during pregnancy starting after the 10th week of pregnancy and thus quite late to monitor correctly the growth pattern. Results from these studies are summarized in Table 1 and briefly reported and discussed below.

In a prospective study, Aharoni et al. examined 32 leiomyomas in 29 pregnant women every 3–8 weeks performing 3–6 ultrasound scans (mean 4.4) for each patient during the course of pregnancy [21]. According to their evaluations, only 22% of uterine leiomyomas grew more than 10% of the initial volume, 19% shrank more than 10% of the initial volume and 59% changed in size by less than 10% of the initial volume thus supporting the idea that most fibroids change shape during pregnancy without changing their volume. The main limitation of this study was that mean gestational age at enrollment was 14.9 weeks of gestation [21]. Neiger et al. who enrolled 72 patients at a similar gestational age reached a similar conclusion [16]. In a contribution by Ozturk et al. including 19 pregnant women, patients were ultrasonographically scanned 2–5 times (3.6 average) during pregnancy with a final examination at the third month postpartum. Timing of evaluation in the first trimester was not well defined. The Authors observed that there was no enlargement in uterine fibroid size in any case as 26% of cases decreased and 74% showed no significant changes. [23]. An increase in fibroid volume was found in 32% of cases with the greatest enlargement occurring prior to 10 weeks of gestation by Rosati et al. that performed ultrasonography at 2–4 week intervals in 36 pregnant women with single leiomyomas. They did not observe a significant volume increase in the second and third trimesters [22]. A retrospective analysis of 107 pregnant women conducted by Hammoud et al. found a decrease in leiomyomas size in 55% of cases between the first and second trimesters (95% CI: 43–66), while an enlargement was found in 45% of cases (95% CI: 34–56). The percentage of uterine myomas that decreased in size between the second and third trimesters was 75% (95% CI: 56–87), while 25% (95% CI: 13–43) enlarged [17]. Lev-Toaff et al. reported a correlation between fibroid size and growth during pregnancy showing that only small leiomyomas increased in size during the second trimester while almost all lesions decreased in size in the last trimester [20].

More recent studies with a particular focus on early pregnancy and performed with better ultrasound technology are consistent for an increase in leiomyoma volume mostly during the first trimester of pregnancy. An observational, longitudinal and prospective study conducted by De Vivo et al. aimed at assessing leiomyoma volume by ultrasound at 11–14, 20–22, and 32–34 weeks of gestation in 38 pregnant women. Considering a change in volume >10% between gestational periods as relevant, 71% of uterine fibroids demonstrated an increased size between the first and second gestational period, and 67% between the second and third trimesters. The logistic regression analysis revealed that the volumetric change occurred mainly in the first half of pregnancy and was influenced negatively by parity and positively by pre-pregnancy BMI, while maternal age correlated negatively with leiomyoma growth over the entire course of pregnancy [13]. Ciavattini et al. examined 109 pregnant women who underwent an ultrasound scan before pregnancy, a second one at 7–8 weeks of gestation, a third examination at 10–13 week of gestation and a fourth scan at 20–22 weeks of gestation [19]. A significant increase for most leiomyomas during the first half of pregnancy (a median growth rate of 122% during the interval between the first and the second scan; median growth rate of 108% between the second and third ultrasound; median growth rate of 25% between the third and fourth ultrasound) without a linear correlation between the growth trend and the gestational age (small leiomyomas seemed to grow at a faster rate in the first trimester and to undergo a slow down by mid-pregnancy) was reported. In contrast with findings by De Vivo et al., at multivariate analysis, these authors could not confirm an association with maternal age, BMI, parity and gravidity. Moreover, these Authors demonstrated a significant positive correlation between hCG serum levels and the diameter of myomas during early pregnancy [19]. In a prospective cohort study of 25 women with fibroids undergoing In vitro fertilization (IVF) conducted by Benaglia et al., pregnant patients were systematically scanned after 4–5 weeks from embryo transfer. A statistically significant leiomyoma enlargement emerged in early pregnancy with a median modification of the diameter of the lesions of +34% (compared with the median modification of +2% in the control group of patients that failed to conceive after IVF) and the median modification of the lesion volume of +140% (+0% in the control group). These results would suggest that uterine leiomyomas may undergo a rapid and remarkable growth during initial pregnancy with a doubling of their volume within 6–7 weeks of gestation [14].

## 3. Sex Steroids in Pregnancy and Leiomyomas

While uterine leiomyoma development and growth are very likely to be dependent on steroid hormones, it cannot be excluded that other hormones and proteins secreted by the fetal, placental and maternal compartments during early pregnancy may have a synergic effect, although the potential influence of all these factors and their combinations have not been systematically investigated yet [24].

### 3.1. Estrogens

Actions of estradiol are mediated by two isoforms of estrogen receptors (ERs): ERα and ERβ. Following binding to the ligand, an ER can mediate a nuclear genomic mechanism, implying direct or indirect binding of the receptor to transcriptional control regions of target genes, or a non-genomic mechanism, starting by receptors at the cell membrane and exerting its effect through kinase pathways [25]. Transcriptional responses to estrogens depend on specific interaction of homo/heterodimeric ERs with estrogen-response elements (ERE) in the promoter regions of target genes. Alternatively, ERs activated by the ligand, can interact with other transcription factor complexes and bind to non-ERE sequences thus regulating several downstream pathways including NF-κB, AP-1 and C/EBPβ [25].

The complexity of estrogen-responsive pathways in the myometrium is mirrored by a constellation of estrogen-related aberrations in leiomyomas. Several studies have demonstrated that levels of ERα and ERβ mRNA transcripts are higher in fibroid cells compared to normal myometrium [26,27,28] although a recent study reported that protein expressions of ERα and ERβ in leiomyoma are similar to those in normal endometrium [29]. Estrogens promote protein and DNA synthesis, increase cell number and induce cell cycle progression in cultured leiomyoma cells [30] but there are also evidence supporting a cross-talk between estrogens and the growth factor signaling in supporting this proliferation. However, one of the major roles of estrogens in the multistep fibroid development appears to be the induction of progesterone receptor expression rendering the leiomyoma tissue more responsive to progesterone signals [31]. Moreover, estrogens can alter the expression of other genes such as *c-fos* and *c-jun*, *connexin 43*, *Insulin-like Growth Factor (IGF)-I*, *IGF receptors* (*IGF-Rs*), and *matrix metalloproteinase-1* [32].

Besides ERα and ERβ, an orphan G-protein-coupled estrogen receptor 1 (GPER1) was detected at the plasma membrane and endoplasmic reticulum. It belongs to the family of 7-transmembrane domain G-protein-coupled receptors and it elicits non-genomic estrogenic action in estrogen-targeted cells. Recently, GPER1 expression was found to be up-regulated in human leiomyoma tissue and primary cell cultures compared to the matched endometrium [33]. Estradiol was indeed shown to increase levels of GPER1 mRNA in fibroid cells but to decrease them in myometrial cells. In the non-genomic pathway, ligand-ER complexes can activate the Ras-Raf-MEK-MAPK signaling pathway and the PI3K/AKT signaling pathway able to regulate several cellular processes at myometrial level including proliferation, survival, and apoptosis [33,34,35,36]. Several molecules of the Ras-Raf-MEK-MAPK pathway have been shown to be overexpressed in fibroids compared to myometrium [37]. Furthermore, estrogens were demonstrated to stimulate phosphorylated protein kinase Cα (PKCα) within a few minutes in both immortalized myometrial and leiomyoma cells. However, MAPK signaling is activated only in the latter suggesting a potential contribution of this rapid pathway to the promotion of leiomyoma proliferation [38].

In pregnancy, estrogens regulate important physiologic processes affecting fetal well-being including progesterone production, utero-placental blood flow, mammary gland development, and fetal adrenal gland activity. In the first months of gestation, estrogen synthesis depends upon androgens present in the maternal bloodstream. A common thinking is that estrogens would be involved in leiomyoma growth during pregnancy mostly because during gestation estrone and estradiol excretion are increased about 100 times over non-pregnant levels and maternal estriol is increased about a thousand-fold. However, although the critical role of estrogens in leiomyoma development is well established, also supported by the reduction of tumor size with a gonadotrophin releasing hormone-based treatment [34], the pattern of these various estrogenic compounds during pregnancy does not mirror the rapid leiomyoma growth during early gestation. 17β-estradiol is produced by the corpus luteum during the first 5–6 weeks of gestation and then by the placenta. Its concentrations are less than 0.1 ng/mL in the follicular phase of menstrual cycle, about 0.4 ng/mL during the luteal phase and then, after fertilization, it shows a sustained rise to a range of 6–30 ng/mL at term pregnancy. Estrone concentrations are less than 0.1 ng/mL during the follicular phase of menstrual cycle and it increases at maximum 0.3 ng/mL during the luteal phase remaining within this range through 6–10 weeks of gestation while its concentration reaches values between 2 and 30 ng/mL at term. Estriol is first detectable at nine weeks; its concentrations reach a plateau at 31–35 weeks; and then increase again at 35–36 weeks [24,26,39].

### 3.2. Progesterone

Originally, it has been observed that the mean mitotic count of fibroid tissue compared to adjacent myometrial tissue was significantly higher in the secretory phase than in the proliferative phase, suggesting that leiomyoma growth was affected by progesterone [40]. The first evidence for the role of progesterone in the pathological proliferation was the demonstration of an increased expression of the progesterone receptor (PR) at both mRNA and protein level in human leiomyomas compared to the adjacent myometrium [41]. Further evidence has derived from in vivo studies performed in a xenograft model obtained with the implantation of leiomyoma tissue in immunodeficient mice. Human leiomyoma xenografts increased in size and volume following estradiol plus progesterone treatment, while estrogen alone was not associated with fibroid growth [31], supporting an essential role of progesterone. Estradiol was however necessary to sustain leiomyoma responsiveness to progesterone by inducing PR expression. The strongest evidence for an in vivo mitogenic effect of progesterone came from clinical trials demonstrating consistently reduced tumor size with antiprogestins treatment (such as RU486, asoprisnil, proellex, and CDB2914) [31,42,43,44].

The effects of progesterone on myometrium and on leiomyomas are mediated by the two main isoforms of PR which is a member of the nuclear hormone receptor superfamily of ligand-activated transcription factors. There are two predominant PR isoforms, named PR-A and PR-B, transcribed by the same gene, with the difference being that PR-B is larger by 164 amino acids. Both isoforms have been shown to be overexpressed in leiomyoma tissue compared to the adjacent myometrium [45,46,47]. Progesterone receptors recognize and bind hormone response elements of target genes and the hormonal ligands induce conformational changes in ligand binding domain helices forming an AF2 pocket that binds to structural motifs within co-regulators serving as mediators of transcriptional activation or repression. These receptors can interact with various transcription factors, such as SP1, AP1, FOXO1 and the p65 subunit of NF-κB [48,49,50,51] to control transcriptional activity of target genes. In leiomyoma cells, PRs can rapidly activate the PI3K/AKT pathway and its effectors, such as p-GSK3β and p-FOXO1 [52]. Moreover, PR-B contains a proline-rich motif that can directly bind to the SH3 domain of SRC kinase thereby activating the ERK signaling pathway [53,54]. In line, R5020, a synthetic progestin acting as a PR agonist, was shown to induce proliferation of leiomyoma cells in vitro, through the phosphorylation of the downstream targets of AKT (FOXO1 and GSK3β) [52].

In addition, there is evidence supporting interactions between progesterone and the growth factor signaling. Both epidermal growth factor (EGF) and its receptor (EGFR) have been shown to be expressed in both myometrial and leiomyoma tissues during the secretory phase of the menstrual cycle but *EGF* mRNA was higher in leiomyoma than myometrium. Interestingly, in monolayer cultures of leiomyoma cells, progesterone was able to increase EGF but not EGFR expression; on the contrary, estrogens increased EGFR expression but did not increase EGF [55].

Progesterone was also shown to down-regulate expression of IGF-I in cultured human leiomyoma cells without affecting the IGF-I receptor expression [56]. Furthermore, a progesterone involvement has been also suggested for leiomyoma expression of transforming growth factor-β type 2, 3 as levels of transforming growth factor (TGF)-β3 were found to be higher during the secretory phase of the menstrual cycle [57,58]. For all these reasons, it is a current opinion that progesterone is at least as important as estrogens for fibroid development [31].

Progesterone concentrations rise from 1–2 ng/mL on the day of the LH surge to a plateau of 10–35 ng/mL in the following seven days. Its concentrations remain within this range from the last menstrual flow to the 10th week of gestation and then embarks on a growth that continues until the term of pregnancy in which progesterone concentrations range from 100 to 300 ng/mL [24].

Overall, as for estrogens, available evidence suggests a crucial role of progesterone in the pathogenesis and growth of fibroids but cannot explain the rapid and impressive growth of these lesions during initial pregnancy.

## 4. Human Chorionic Gonadotropin in Pregnancy and Leiomyomas

Human chorionic gonadotropin stimulates progesterone production, decidualization, angiogenesis and cytotrophoblast differentiation and for these reasons it represents a critical factor for the initiation and maintenance of early pregnancy. hCG belongs to a family of glycoprotein hormones which includes LH and Follicle Stimulating Hormone. While α-subunits are similar, β-subunits are different among the glycoprotein hormone members. The β-subunits of hCG and LH are highly homologous and both LH and hCG activate the same LH/hCG receptor. hCGβ contains 145 amino acids while LHβ contains 120 amino acids. The C-terminal peptide comprising amino acids 121–145 is unique to hCG while the core regions of LH and hCG comprising amino acids 1–120 are highly similar, the homology between these being about 85%. All human tissues appear to produce hCG, but only the placenta can glycosylate the protein favoring the addition of components such as fructose, galactose, mannose, galactosamine, glucosamine and sialic acid. This results in the reduction of its rate of metabolism and in an increased biologic activity through a long half-life. Although the other sugars are important for hormonal function, the determinant of the biologic half-life is represented by sialic acid. There are five variants of hCG, each having identical amino acid sequence: hCG, sulfated hCG, hyperglycosylated hCG, hCG free β-subunit and hyperglycosylated-free β-subunit [59]. hCG contains 30% sugar by molecular weight, hyperglycosylated hCG contains 39% sugar and hyperglycosylated free β-subunit contains 42% sugar by molecular weight.

All the variants are present in the maternal blood. Moreover, the glycosylation level of hCG varies throughout pregnancy, with increased glycosylation and production of normal molecules in early gestation when the biologic actions of hCG are critical [60]. Hyperglycosylated hCG is thought to favor implantation of trophoblasts deep into the myometrium and to stimulate cytotrophoblast growth while hCG has been shown to promote the fusion of cytotrophoblast cells to syncytiotrophoblast cells and to drive angiogenesis of the spiral arteries [59].

The *hCG* gene is expressed in both cytotrophoblasts and syncytiotrophoblasts, but the hCG synthesis is mainly in the syncytiotrophoblasts. Production and secretion of hCG during pregnancy are thought to be the result of complex interactions among sex steroids, gonadotrophin releasing hormone (GnRH), cytokines, and growth factors. hCG regulation is negatively affected by inhibin and progesterone. The direct inhibition by this hormone could explain the lower levels of serum hCG after the 10th week of gestation when placental progesterone production increases [61]. Conversely, GnRH stimulates the secretion of hCG and a similar behavior can be demonstrated in response to other peptides, such as interleukin-1β [62]. Inhibin restrains whereas activin enhances the GnRH-hCG system, with a positive influence of estrogens and a negative effect by progesterone [63]. By binding to activin, follistatin prevents the stimulatory activity of activin. Other growth factors, particularly IGF-I and tumor necrosis factor-α (TNF-α) also influence hCG secretion.

Human chorionic gonadotropin blood levels undergo a rapid exponential increase in the first weeks of pregnancy, doubling in concentration every 2–3 days in the first trimester of gestation. The peak of hCG levels occurs at 10 weeks of gestation with concentrations of about 110,000 mIU/mL. Then they decrease rapidly between 12–16 weeks of gestation halving their concentration every 2.5 ± 1 days and continuing to fall from 16 to 22 weeks at a slower rate halving their concentration every 4 ± 2 days to became around 10% of first trimester values [24].

It is the rapid exponential raise in serum hCG at the beginning of gestation and the remarkable nonlinear growth of small fibroids during the first weeks that lead to the clinical speculation that hCG may have a potential role in the striking growth of leiomyomas during initial pregnancy.

### 4.1. Luteinizing Hormone/hCG Receptors in Myometrium and Leiomyomas

Human chorionic gonadotropin acts by binding to LH/hCG receptor, a G protein coupled receptor with a characteristic structure with seven transmembrane domains. It is anchored in the plasma membrane by two palmitoylated cysteine residues in the C-terminal tail.

The LH/hCG receptor in myometrium was identified in pigs, in rabbit and it has to be underlined that most of the available data on the receptor expression, regulation and signaling pathways refer to animal studies [64,65,66,67,68,69]. In all the species studied, in the myometrium as well as in the endometrium and fallopian tubes, expression of the receptor was higher in the secretory phase of the cycle compared to the follicular phase [70,71]. In humans, the receptor is also expressed in the myometrium during pregnancy and is down-regulated following the onset of labor [69]. Transcripts for LH/hCG receptor in leiomyomas were first demonstrated in 1995 by northern blotting and the presence of the protein was confirmed by western blotting [72]. In situ hybridization and immunocytochemistry revealed that smooth muscle cells, and not the connective tissue, in leiomyomas and corresponding normal myometria contained the LH/hCG receptor mRNA transcripts and proteins. However, the receptor levels were lower in leiomyomas than in corresponding normal myometria. Finally, the presence of *LH/hCG* receptor mRNA was confirmed in both myometrial and leiomyoma cells by PCR [73]. Overall, the presence of functional LH/hCG receptors in fibroids has been repeatedly confirmed, thus further supporting the intriguing possibility that hCG can affect leiomyoma growth.

**Figure 2:**
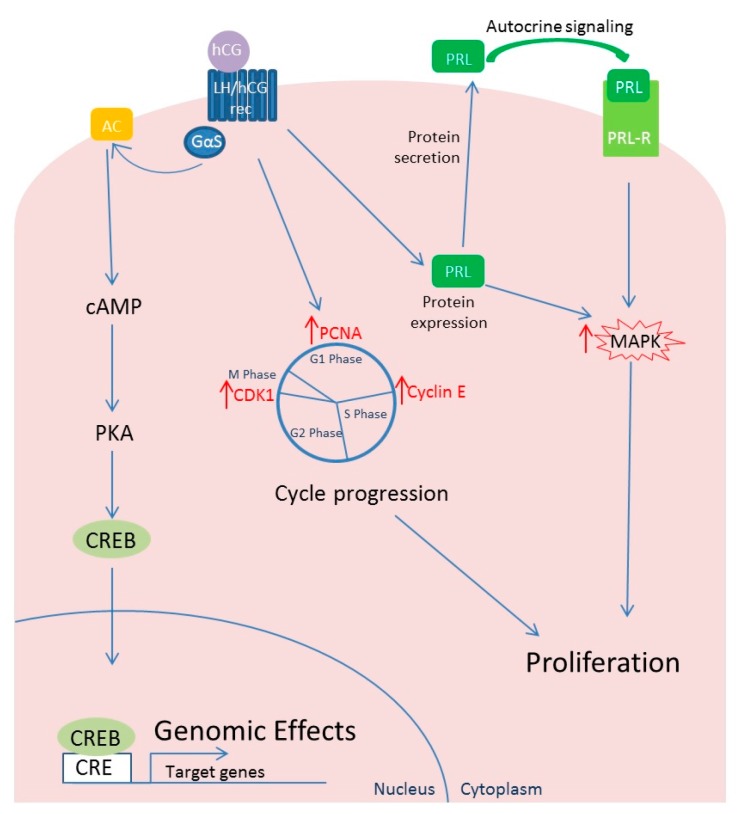
hCG signaling and effects on leiomyomal cells. In human myometrial smooth muscle and leiomyomas cells, hCG: (I) activates the cAMP/PKA signaling pathway causing changes in the expression of several target genes mediated by related transcription factors such as CREB; (II) promotes cell cycle, increasing expressions of key proteins like PCNA, cyclin E and cdc2; and (III) promotes production of prolactin that, directly and through autocrine/paracrine effect, increases cell proliferation. The signaling pathways regulating cell cycle progression genes in leiomyomal cells have not been clarified yet. hCG: human Chorionic Gonadotropin; LH: luteinizing hormone; LH/hCG rec: LH/hCG receptor; AC: Adenylate cyclase; PKA: protein kinase A; CREB: cAMP Responsive Element Binding Protein; CRE: cAMP Responsive Element; PCNA: Proliferating cell nuclear antigen; CDK1: cyclin-dependent kinase 1; PRL: Prolactin; PRL-R: Prolactin Receptor; the red arrows represent increased expression.

Human chorionic gonadotropin generally activates two different signaling systems: adenylate cyclase and phospholipase C (PLC) [74]. Levels of expression of the adenylyl cyclase stimulatory G protein, Gαs, substantially increase within the myometrium during gestation [75]. As a consequence of adenylyl cyclase activity, increased cAMP formation and activation of protein kinase A (PKA) cause changes in the expression of several genes through activation of transcription factors interacting with the palindromic cAMP response elements (CRE) in the promoter regions of target genes [76] (Figure 2). Although PLC has been identified as mediator of hCG effects in several human tissues [74], in a dated study, Kornyei and colleagues [67] demonstrated that PKA inhibitors, but not PKC inhibitors, were able to revert the hCG-mediated effect on human myometrial smooth muscle cells. This evidence would suggest that the PLC/PKC signaling activated by Gαq is not involved as mediator of hCG action in human myometrium cells. Unfortunately, the literature in this regard is too scant to confirm or confute this evidence.

### 4.2. Effects of hCG on Leiomyoma Cells 

Human chorionic gonadotropin has been shown to increase fibroid cell number both directly and through an autocrine/paracrine effect mediated by prolactin secretion [67,77,78]. In fact, it has been known for many years that hCG is able to stimulate in vitro proliferation of human myometrial smooth muscle cells [67]. The administration of different concentrations of hCG (from <1 to 100 nM) to myometrial smooth muscle cells in culture was able to induce an increase in cell density reaching a maximum at about 30 nM followed by a decline. In addition, prolactin, EGF and growth hormone (GH) were shown to increase the myometrial muscle smooth cell density but only EGF was more effective than hCG. Estradiol and progesterone did not have an additive effect on hCG action (Figure 2).

Horiuchi and coworkers have investigated cell proliferation and the expression of cell cycle-related proteins in normal uterine smooth muscle and uterine leiomyoma cells to assess the direct effect of hCG showing that, even at a low concentration, the molecule could exert a direct influence over the growth of uterine fibroids by promoting cell cycling [73]. Although hCG could increase the number of viable cells in both cell types, cell number in hCG-treated cultures was significantly greater for leiomyoma samples than for myometrial samples on Day 3 of culture but not after six and nine days of culture. Treatment of leiomyoma cells with hCG (30 nm/L) determined an up to 500% increase in the cell number after three days. Supporting the idea that hCG may exert a direct effect over the growth of uterine leiomyomas by regulating cell cycle control mechanisms, the expressions of proliferating cell nuclear antigen (PCNA), cyclin E and cdc2 were all significantly increased in leiomyoma cells upon hCG treatment (Figure 2). Particularly, the expressions of cyclin E and cdc2 were both significantly increased even at the lowest concentration (3 nmol/L) of hCG used, although the molecular mechanisms mediating this action are not yet completely established. In myometrial cells, an increase in protein expression was observed for cyclin E and cdc2 only for the highest concentration (30 nmol/L). Cdk2 expression was conversely not affected by hCG treatment of leiomyoma cells [73,79,80].

Human myometrium and leiomyoma synthetize and secrete prolactin but leiomyomas have been shown to secrete larger amounts of prolactin in vitro than normal myometrial tissue [81]. Prolactin secretion can be stimulated by hCG treatment of leiomyoma cultures [77]. Prolactin receptor mRNA was found to be expressed in human leiomyoma cells at levels comparable to those in term decidua and prolactin has been shown to have a mitogenic effect on leiomyoma-derived smooth muscle cells at a dose from 5 to 50 nM causing a rapid phosphorylation of the MAPK signaling pathway [78,82]. Interestingly, when genes differentially expressed in genetically altered leiomyomas compared to the corresponding myometrial tissues were evaluated, ingenuity pathway analysis revealed a significant activation of the prolactin signaling in leiomyomas. Prolactin was one of the most up-regulated genes in leiomyomas with *HMGA2* rearrangements, *MED12* mutations and *COL4A5*-*COL4A6* deletion supporting its role as a mitogenic autocrine growth factor in human leiomyoma tumorigenesis.

### 4.3. Differential Behavior of LH and hCG in Relation to Leiomyoma Development

Although LH and hCG have similar structures and act on same receptor [74], they display peculiar characteristics: LH is produced in a pulsatile fashion and its half-life is of 60–120 min [83], while hCG is produced in an increasing and non-pulsatile manner [84] and shows half-life of several hours. In addition, being structurally different, the interaction of these hormones with the same receptor is different [85]. LH/hCG receptor belongs to the leucine-rich-repeat-containing G-protein coupled receptor subfamily with an extracellular domain containing several leucine-rich repeats [86], responsible for the recognition of glycoprotein hormones. hCG binds this domain with higher affinity than LH [87]. Upon hormone binding, depending on ligand, the receptor fundamentally differs in patterns of expression of regulated genes and downstream effects [88,89,90]. However, this panorama does not fully describe the complexity of cascades activated by these two hormones because hCG and LH can act on same signaling pathways with a different impact [74]. The behavior of the receptor in leiomyomas in response to the two hormones is only barely known and seems to be quite different between LH and hCG. In a dated study, based on known proliferative effects on uterine smooth muscle cells by hCG [73] and the high level of homology between hCG and LH, Baird and colleagues investigated the leiomyomata development in response to LH in perimenopausal women. Urine samples were collected and LH concentration measured by immunofluorometric assay. A significant association was demonstrated between medium-high LH levels and presence of fibroids, in particular with large tumors (≥4 cm). However, in contrast to what previously observed for hCG, high urinary LH concentrations were found to significantly correlate with earlier leiomyomata onset and not with leiomyomata growth [91].

Both LH and hCG levels increase in perimenopause [90,92] partly explaining the common sudden growth of fibroids in this particular phase of reproductive life [93]. Therefore, clarifying if and how these two hormones act in the onset and/or development of leiomyomas in perimenopause would be of great interest also to better understand their roles in pregnancy. The affinity to the receptor and/or the pattern of action might have a critical role.

## 5. Cross-Talk between hCG and Other Pathways in Leiomyoma Development

### 5.1. Transforming Growth Factor (TGF)-β and hCG

TGF-β is a ubiquitous cytokine involved in cell growth and differentiation and ECM production. Four isoforms of TGF-β (called 1–4) are present in humans. TGF-β is secreted as latent protein and, after an enzymatic reaction, an active form is released. The active TGF-β ligand binds available TGF-β receptors. There are three TGF-β receptors (Tβ-RI, Tβ-RII and Tβ-RIII) belonging to the single-domain transmembrane glycoproteins family. Tβ-RI and Tβ-RII have short extracellular domains and a long cytoplasmic region with serine/threonine kinase activity. Once TGF-β binds directly the constitutively active kinase domain of Tβ-RII, this complex is recognized by the Tβ-RI, assembling a heterodimer (Tβ-RI:Tβ-RII) in which the Tβ-RII phosphorylates the Tβ-RI, initiating its kinase activity [94]. Once activated, Tβ-RI recognizes and phosphorylates Smad transcription factors, starting the signaling cascade [95]. Tβ-RIII is a membrane-anchored proteoglycan protein and is not a direct mediator of TGF-β signal transduction [96].

Human leiomyoma express mRNA and proteins for TGF-β isoforms 1–3 and their receptors [97]. In particular, TGF-β3, Tβ-RI and Tβ-RII are overexpressed in human leiomyoma compared with adjacent myometrium [98,99,100,101]. In a co-culture system with human leiomyoma cells and uterine leiomyoma-derived fibroblasts, an increased secretion of TGF-β1 and TGF-β3 in the medium, an augmented number of leiomyoma cells and an elevated deposition of ECM proteins have been observed compared to a culture of human leiomyoma cells alone, highlighting the key role of soluble factors and of the ECM components in tumor progression [102]. Many investigators have demonstrated the activation of the TGF-β pathway through activation of Smad transcription factors in leiomyoma tissue and cells [103,104,105]. An increase of the active phosphorylated form of both Smad3 and Tβ-RII has been found in leiomyoma tissue compared to the matched myometrium in the setting of largely equivalent levels of unphosphorylated total Tβ-RII [105].

Recently, several LH/hCG receptor-independent hCG activities have been demonstrated, including activation of the Tβ-R by some hCG variants [106,107,108]. Hyperglycosylated hCG and hCG free β-subunit have been shown to be able to bind Tβ-RII in cells producing these hCG forms [108,109,110]. This evidence is supported by the three-dimension analysis of hCG molecules highlighting a structural similarity between the hCG β-subunit and a subunity of TGF-β [111]. In addition, the exposition of endothelial and aortic smooth muscle cells to hyperglycosylated hCG induces phosphorylation of Smad2 and a genomic activation of Smad-responsive elements [110].

The association between hCG and cancer is well-known in some human tumors (such as bladder, cervical, colorectal, endometrial, lung, ovarian, pancreatic and vulvar cancer) and therefore it has been proposed a mechanism for which hCG free β-subunit and hyperglycosylated hCG, binding Tβ-R, would promote growth of some cancer cells and ECM proteins deposition [58]. This mechanism, in light of the above observations, may be true also for leiomyoma growth and needs to be further investigated.

### 5.2. Vitamin D and hCG 

A protective effect of vitamin D on uterine leiomyoma has been suggested and is biologically plausible as the vitamin D receptor (VDR) has been shown to be expressed in both myometrial and leiomyoma tissues. The active form of the vitamin D can indeed exert a growing-inhibiting effect on both myometrial and leiomyoma cells. In line, in in vitro experiments and in an in vivo model of fibroids in rats, the active form has been shown to cause a strong reduction of the mass growth [112,113,114,115]. Finally, serum concentrations of 25-hydroxyvitamin-D_3_ [25(OH)D] have been consistently reported to be significantly lower in affected versus not affected women [116,117,118]. A possible mechanism through which 1,25-dihydroxyvitamin-D_3_ [1,25(OH)_2_D] can function in uterine fibroid cells is through the induction/activation of the nuclear VDR levels, which can interact with retinoid X receptor-α (RXRα) to form a VDR–RXRα complex. This VDR–RXRα heterodimeric complex further binds to specific vitamin D responsive elements in the promoter regions of vitamin D target genes.

**Figure 3:**
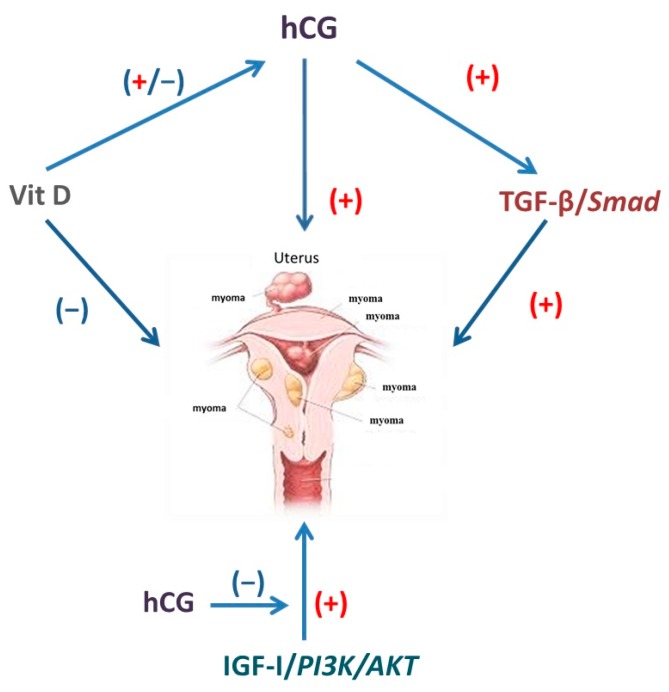
Potential mechanisms of hCG action on leiomyomal cells. hCG: (I) may activate the TGF-β/Smad pathway; (II) may interact with the Vitamin D signaling; and (III) may affect the IGF-I-induced cell proliferation. hCG: human chorionic gonadotropin; Vit D: vitamin D; TGF-β: transforming growth factor-β; IGF-I: insulin growth factor I.

A cross-talk between the vitamin D pathway and the hCG-mediated effect has been demonstrated in cultured human trophoblasts where a direct regulatory effect of 1,25(OH)_2_D on hCG production has been demonstrated (Figure 3). The active form of vitamin D was shown to regulate hCG in a time-dependent manner, changing hormone expression and secretion. A stimulatory effect was observed after 6 h of treatment probably due to the rapid 1,25(OH)_2_D-dependent increase in intracellular cAMP since, after 10 min of treatment, intracellular cAMP content significantly increased in a dose-dependent manner. 1,25(OH)_2_D also up-regulated *β-hCG* mRNA [119,120]. Preincubation of cells with a selective inhibitor of PKA could prevent this 1,25(OH)_2_D-dependent protein and gene expression stimulation of hCG detected at the 6 h incubation period. The stimulatory effects of 1,25(OH)_2_D were not further evident after 24 h, and, after two consecutive days, the effects were rather inhibitory. Inhibition was evident at mRNA level after 24 h treatment and later for hCG protein. This repressive effect by 1,25(OH)_2_D could not be attributed to decreased cell viability, since, under the same conditions, 1,25(OH)_2_D up-regulated *CYP24A1* gene expression [121].

The demonstration of genomic-mediated effects of 1,25(OH)_2_D on hCG would suggest that the *hCG* gene promoter have VDR-dependent regulatory regions. Five putative VDR/RXR heterodimer binding sites in the *hCGβ-5* gene promoter [122], which probably may be acting as 1,25(OH)_2_D dependent-transcriptional regulatory regions, have been reported.

### 5.3. Insulin-Like Growth Factor-I and hCG 

A potential mediator of steroid hormone effects of on uterine fibroids is IGF-I, a small polypeptide that is produced by many tissues and that functions acting via endocrine, paracrine and autocrine pathways to regulate proliferation and apoptosis in target cells [123]. The biological functions of IGF-I are mainly mediated by the type-I IGF-receptor (IGF-IR), acting through the PI3K/AKT pathway (Figure 3) [124].

In the normal myometrium and in the other uterine tissues, both IGF-I and the IGF-IR are co-expressed [125,126]. Two studies reported that fibroids bind more IGF-I than normal myometrium where the factor is thought to act to promote leiomyoma growth in an autocrine/paracrine fashion [127,128]. Indeed, IGF-I has been shown to increase significantly the expression of immunoreactive PCNA and to stimulate cell proliferation in cultured leiomyoma cells compared to untreated controls [129].

The role of the IGF-I pathway in leiomyoma development has been well studied in a rodent model for this disease, the Eker rats in which leiomyomas are hormonally responsive tumors derived from a mutation in the *Tsc-2* tumor suppressor gene [130]. Non-carrier isogenic Eker females were used to determine the status of IGF-I signaling components in the normal myometrium while tumor-bearing carrier females were used for analyses concerning leiomyomas. A dysregulated expression of IGF-I was found in leiomyomas resulting in mRNA levels that were 7.5-fold greater than those seen in age-matched normal myometrium. Increased IGF-I expression in leiomyomas correlated with increased tyrosine phosphorylation of signal-transducing protein insulin receptor substrate-1. These data would suggest that overproduction of IGF-I by these tumors would result in activation of signaling pathways emanating from the IGF-IR.

Evidence for a regulation of IGF-I and its receptor by hCG in leiomyomas is lacking. However, the ability of hCG to affect the IGF-I pathway has been demonstrated in luteinized granulosa cells (Figure 3). Both IGF-I and hCG are known to be involved in the regulation of proliferation of luteinized granulosa cells, the first with a stimulatory manner activating the PI3K/AKT pathway and the latter via an inhibitory action, attenuating the activity of AKT and abolishing the IGF-1-induced cell proliferation [131]. Expression of IGF-1R was not affected by hCG.

From these previous experiments, we can suppose a possible role carried out by the molecular interactions involving the pathway hCG-IGF-1 on leiomyoma cells during their proliferation in concomitance of genetic factors risk and other pathways.

## 6. Conclusions and Prospects

The role of hCG in pregnancy-related fibroid growth has been up to now neglected. The simplistic view ascribing an essential role exclusively to sex steroids lacks solid bases. Conversely, there is a large body of literature showing direct and indirect effects of hCG. Further evidence is however required to fully elucidate this intriguing and complex molecular effect. The definitive clarification of the precise role of hCG may shed light on some intriguing but poorly explained clinical observations.

Firstly, we deem of particular relevance disentangling why the growing effect of hCG is short-lasting and tends to disappear after the first few weeks of pregnancy. One may speculate that the LH/hCG receptor is exclusively sensitive to exponential raises and thus, once hCG terminates its rapid growth, the stimulating effect vanishes. Noteworthy, a similar mechanism was documented for corpora lutea at the beginning of pregnancy. The exponential rise of hCG is actually essential to rescue corpora lutea. This may involve mechanisms such as receptor down-regulation or prolonged receptor occupancy. Albeit acting on the same receptor, LH and hCG may have profound dynamic differences [132]. This possibility remains however speculative. Experimental evidence linking directly fibroid growth to exponential hCG growth is required.

Secondly, based on the role of hCG in regulating fibroid growth, one should better evaluate whether LH may have similar effects. In particular, it was hypothesized that the highly fluctuating serum LH levels that typically occur in perimenopause (mimicking the hCG raise in early pregnancy) may also affect fibroid growth and may thus explain the common sudden growth of fibroids in this particular phase of reproductive life [91]. The possible impact on such evidence in clinical management would be relevant since it would justify new simple prophylactic approaches (such as the use of estroprogestins) to prevent perimenopause-related growth and thus avoid surgery. On the other hand, this possibility contrasts with the shrinkage of leiomyomas in menopause, i.e., a condition characterized by elevated levels of LH as a consequence physiological depletion of ovarian function. It may be speculated that the concomitant presence of steroid hormones might be essential for the LH effect on target receptors. In other words, LH could not display its detrimental effects on leiomyomas growth in the absence of steroids. Moreover, we cannot exclude that other hormones may be concomitantly involved and that their effects might be additive. Actually, defining whether LH is causal or a marker for susceptibility will require further research.

Finally, it is worth mentioning that a neglected but fascinating body of literature showed a regression of fibroids after delivery [133,134]. Most importantly, some fibroids that were present before conception actually disappeared after delivery and puerperium. Some authors speculated that the sudden growth during pregnancy may ultimately cause apoptotic phenomena once the growing stimuli are off and the fibroids are exposed to the devastating local effects that are associated to uterus involution after delivery. Interestingly, a similar pattern, i.e., rapid growth during pregnancy and subsequent disappearance has been also shown for decidualized endometriomas [135,136,137] and prolactinomas [138]. We may actually suppose that the growth of leiomyomas in early pregnancy could be a multifactorial mechanism involving steroids, local growth factors and also other placental hormones, suggesting a combination of complexity and redundancy of molecular pathways. Disentangling the molecular and biological mechanisms regulating these processes may open new avenue of research and possible innovative therapeutic approaches.

## Figures and Tables

**Table 1:** Pattern of growth of uterine leiomyomas during pregnancy, cases reported in literature.

Author, Year	*n* of Cases	I Trimester (%)	II Trimester (%)		III Trimester (%)
Lev-Toaff et al. (1987) [20]	71	+	+ (30); − (15) for small fibroids/* + (14); − (48) for large fibroids		− (35) for small fibroids/* − (59) for large fibroids
Aharoni et al. (1988) [21]	29	NR	NS		NS
Rosati et al. (1992) [22]	36	+ (32) *	NS		NS
Neiger et al. (2006) [16]	72	NR	NS		NS
Hammoud et al. (2006) [17]	107	− (55); + (45) *		− (75); + (25) *	
Ozturk et al. (2009) [23]	19	NR	NR		NR
De Vivo et al. (2011) [13]	38	+ (71) *		+ (67) *	
Benaglia et al. (2014) [14]	25	++ *	NR		NR
Ciavattini et al. (2016) [19]	109	++ *	+ *		NR

+, increase in volume; −, decrease in volume; NS, not significant volume changes; NR, not reported; *, statistically significant. Numbers in parentheses represent the percentage of fibroids that increase/decrease in volume.
